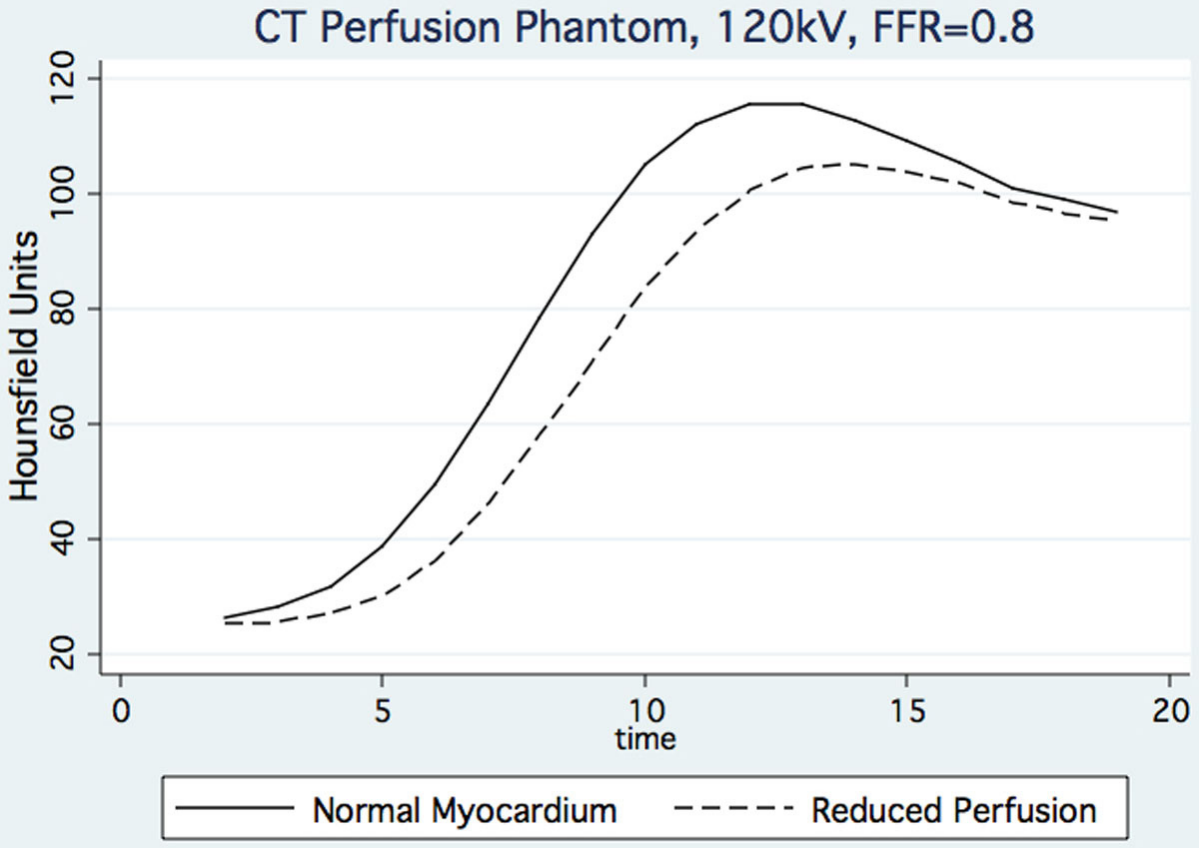# Direct comparison of MR and CT perfusion utilizing a myocardial perfusion phantom

**DOI:** 10.1186/1532-429X-14-S1-P218

**Published:** 2012-02-01

**Authors:** James Otton, Amedeo Chiribiri, Geraint Morton, Boris Bigalke, Matthias Paul, Shazia T Hussain, Roy Jogiya, Eike Nagel

**Affiliations:** 1 Imaging Sciences, King's College London, London, UK; 2 University of New South Wales, Sydney, NSW, Australia

## Background

Myocardial CT perfusion is a relatively new technique which enables the assessment of perfusion deficits through first pass imaging in combination with pharmacological stress. Comparison of CT and MR perfusion has been limited and in-vivo assessment is affected by physiological variability, acquisition timing during first-pass contrast perfusion and parameter selection. We utilized a myocardial perfusion phantom to precisely compare high resolution k-t SENSE MR perfusion with single phase CT perfusion under identical imaging conditions.

## Methods

We utilized a customized MRI and CT compatible myocardial perfusion phantom to represent the human circulation. The phantom includes venous input, cardiac chambers, pulmonary and aortic output, coronary arteries and two diffusion chambers to represent the myocardium. Coronary flow and myocardial perfusion may be precisely controlled. The phantom has been extensively validated within the MR environment and CT upslope and myocardial perfusion values closely match published data as well as in-vivo experimental results. CT perfusion studies were performed with a Philips iCT (256 slice) CT, with isotropic resolution of 0.6mm^3^ and contrast dosing according to clinical protocols. One volumetric acquisition of the myocardial volume was performed each second. MR perfusion was performed using a 3T Philips Achieva TX system, equipped with a 32-channel cardiac phased array receiver coil. We used a saturation recovery gradient echo method (repetition time/echo time 3.0ms/1.0ms, flip angle 15°; effective k-t SENSE acceleration 3.8, spatial resolution 1.2x1.2x10 mm. Noise estimates were based on published clinical data.

## Results

Contrast by CT perfusion is highly dependent on photon energy and to a lesser extent on timing of CT acquisition within the myocardial perfusion upslope. For a differential myocardial perfusion equivalent to an FFR of 0.8, the peak contrast to noise (CNR) within individual images at 120kV CT was 1.05 vs 0.77 for k-t-MR perfusion. While contrast increases with reduced photon energy the CNR is effectively unchanged at 100kV and 80kV due to increased noise, although there is reduced associated radiation dose. Perfect timing of a single acquisition during myocardial contrast inflow is difficult to achieve and CNR by CT decreases to 0.68-0.84 two seconds from peak contrast. Both techniques are subject to motion and other artifact while noise is dependent on body morphology, as well as imaging parameters.

## Conclusions

For an identical myocardial perfusion deficit, the single image contrast to noise ratios generated by CT and KT-accelerated MR are similar. While single phase CT perfusion offers higher spatial resolution, MR perfusion allows multiple time point sampling and quantitative analysis.

## Funding

St Vincent's Clinic Foundation.

National Health and Medical Research Council of Australia.

Wellcome Trust and EPSRC grant number WT 088641/Z/09/Z.

**Figure 1:**